# A large animal lung cancer model for the evaluation of intratumoural cisplatin delivery strategies

**DOI:** 10.3389/fonc.2026.1906629

**Published:** 2026-10-09

**Authors:** James Meehan, David Collie, Ziyuan Chang, Rachael Gregson, R. Eddie Clutton, Stephen N. Greenhalgh, Lynn Grant, Sara Kennedy, James Nixon, Chris Cousens, Helen Todd, Jennifer Savage, Jo Moore, David J. Griffiths, Lorna J. Eades, Gavin Sim, Nicole Storer, Chad E. Eckert, Mark Gray

**Affiliations:** 1 The Royal (Dick) School of Veterinary Studies and Roslin Institute, University of Edinburgh, Edinburgh, United Kingdom; 2 Moredun Research Institute, Pentlands Science Park, Penicuik, United Kingdom; 3 The School of Chemistry, University of Edinburgh, Edinburgh, United Kingdom; 4 The School of Geoscience, University of Edinburgh, Edinburgh, United Kingdom; 5 Interventional Oncology, Johnson & Johnson Enterprise Innovation, Inc., New Brunswick, NJ, United States

**Keywords:** cisplatin, experimental animal models, lung neoplasms, ovine, translational medical research

## Abstract

**Introduction:**

Cisplatin has been a standard chemotherapeutic agent for advanced non-small cell lung cancer for decades. Conventionally administered intravenously, it undergoes systemic distribution, exposing both tumour and healthy tissues to the drug. Endobronchial ultrasound-guided transbronchial needle injection of cisplatin is emerging as a potential intratumoural drug delivery (ITDD) strategy. However, rigorous preclinical research is needed before ITDD can be more widely adopted.

**Methods:**

We used our experimentally induced ovine pulmonary adenocarcinoma (OPA) model, generated by Jaagsiekte sheep retrovirus (JSRV) infection, to evaluate transthoracic and transbronchial ITDD techniques. We compared systemic platinum exposure following intravenous cisplatin delivery with that following intratumoural cisplatin administration. Both non-recovery and recovery studies were conducted in a total of eight sheep.

**Results:**

ITDD techniques were successfully developed and performed in three tumour bearing sheep during non-recovery procedures. To provide a reference for systemic platinum exposure, intravenous cisplatin was administered to three JSRV-infected sheep that did not develop OPA. Intravenous administration produced a rapid plasma platinum peak followed by a rapid decline. ITDD resulted in preferential platinum deposition within targeted lung tumours while producing substantially lower systemic platinum exposure compared with intravenous administration. Intratumoural cisplatin delivery was also evaluated in a recovery procedure using a single sheep, comparing single-site and fractionated administration over a 28-day period, with tumour biopsies confirming lesion presence before treatment. Following ITDD, tumour volumes were observed to decrease over the study period. The recovery experiments demonstrated the feasibility of repeated tumour biopsies, intratumoural injections, longitudinal CT monitoring and recovery anaesthesia using the OPA model.

**Discussion:**

To our knowledge, this is the first report of direct intratumoural cisplatin administration into ovine lung tumours. This study also provides proof-of-concept that this clinically relevant large animal lung cancer model can serve as a translational platform for evaluating intratumoural therapeutic delivery strategies relevant to human lung cancer.

## 1 Introduction

Worldwide, human lung cancer is the most frequently diagnosed cancer, with ~2.5 million new cases and ~1.8 million cancer-related deaths occurring per year (1). Despite our improved understanding of the pathogenesis of this disease, surprisingly few novel therapeutics have reached the clinics. Cisplatin (cis-diamminedichloroplatinum) has been used as a chemotherapeutic agent since its approval for use in the Unites States of America in 1978 (2), and is still considered standard-of-care treatment for advanced non-small cell lung cancer (NSCLC) (3). The standard route of cisplatin administration is intravenous, which results in systemic distribution and consequently exposes both tumour and normal tissues to cisplatin, thereby increasing the risk of dose-limiting toxicities in non-tumour tissues (4), often limiting its clinical use. As a result, there is increasing interest in localised delivery strategies that may permit higher intratumoural drug concentrations and reduced systemic toxicity, thereby enhancing the therapeutic index of cisplatin (5–7).

Recently, endobronchial ultrasound-guided transbronchial needle injection of cisplatin has emerged as a potential intratumoural drug delivery (ITDD) technique for patients, either as a neoadjuvant treatment (8), or for patients who have failed prior radiotherapy and are not undergoing concurrent systemic treatment (9–11). While ITDD represents a promising strategy to enhance therapeutic efficacy and reduce systemic toxicity, this procedure is currently reserved for experimental settings or clinical trials. Further preclinical and clinical studies are required before ITDD can be considered for broader clinical application in the treatment of lung cancer.

Although murine models are commonly used in preclinical lung cancer studies, there is increasing recognition of the limitations inherent to these models in replicating the complexity of human disease (12). Consequently, there is growing consensus that translational research employing comparative large animal models, which more closely recapitulate human tumour biology and treatment response (13–15), are essential for advancing novel therapies toward clinical implementation (16, 17). Indeed, recent publications focusing on localised lung tumour treatment have stated the need for standardised large animal models of lung tumours for the testing of new therapies (18). Several large animal models have been developed to facilitate the evaluation of locoregional lung cancer therapies. These include transbronchial cryoablation studies performed in healthy porcine lungs to optimise bronchoscopic therapeutic delivery (18), as well as genetically engineered porcine models that develop orthotopic pulmonary neoplasia and provide opportunities to investigate tumour biology (19, 20). However, relatively few large animal models combine clinically relevant orthotopic lung tumours with longitudinal imaging, repeat biopsy acquisition and image-guided therapeutic intervention within the same platform.

Because of the close anatomical and physiological similarities between the ovine and human lung (21), sheep have long been recognised as a valuable large animal model for human respiratory diseases (22) and for the development of pulmonary therapeutic strategies (23). Both species possess comparable lung size, airway branching architecture, respiratory epithelial cell distribution, and pulmonary physiology (22, 24, 25), enabling the use of clinically relevant diagnostic and interventional procedures in sheep. Importantly, the size of the sheep lung permits surgery, advanced imaging, ultrasound, bronchoscopy and longitudinal therapeutic monitoring using equipment and techniques analogous to those employed in human patients (23, 26–29). These procedures cannot readily be performed in murine models, making sheep particularly valuable for investigating respiratory disease pathogenesis and evaluating pulmonary therapeutic and drug-delivery strategies prior to clinical translation (23). Combined with their suitability for repeated sampling and long-term studies, these characteristics have established sheep as important translational models for a range of respiratory diseases, including asthma (30), chronic obstructive pulmonary disease (31), respiratory viral infections (32), and, more recently, lung cancer (33). Altogether, these capabilities establish sheep as valuable large animal models for the development and evaluation of image-guided lung cancer therapies and other translational respiratory interventions.

Importantly, naturally occurring diseases in sheep can also serve as relevant models for human lung cancer research. One such disease, ovine pulmonary adenocarcinoma (OPA), is a contagious neoplastic lung disease caused by Jaagsiekte sheep retrovirus (JSRV). A detailed comparison of OPA and human lung adenocarcinoma, including pathological, molecular and translational similarities and limitations, has been reviewed previously (21, 34). OPA exhibits several key similarities to specific subtypes of human lung cancer, including histopathological features, gene expression profiles, and the activation of oncogenic signalling pathways (33, 35). Indeed, OPA is now regarded as a robust and translationally relevant model for certain forms of human lung cancer (21, 33, 35). Early OPA lesions resemble minimally invasive or lepidic-predominant adenocarcinoma, while typical advanced lesions are more akin to adenocarcinoma with acinar-predominant or papillary growth patterns. Overall, OPA is most similar to the rare multifocal, non-invasive mucinous form of human lung adenocarcinoma (21). OPA therefore represents an excellent One Health comparative oncology model, whereby investigation of a naturally occurring veterinary disease has the potential to improve both animal health and human lung cancer research through shared biological mechanisms, pathological features and therapeutic strategies. The development and application of such comparative models relies on close collaboration between veterinary pathologists, oncologists, imaging specialists, surgeons and translational scientists, ensuring that pathological characterisation and therapeutic evaluation remain closely aligned with clinically relevant questions in both veterinary and human medicine.

Our group has recently developed an experimental infection model capable of generating localised gross OPA lesions after either bronchoscopic catheter instillation or intraparenchymal injection of JSRV into a segmental lung lobe (68, 27). Through ultrasound and computed tomography (CT) scanning, we are able to track OPA development after JSRV inoculation. Using this model, the aims of this study were to (i) establish and evaluate a novel experimental platform for ITDD using both endobronchial and transthoracic injection techniques, and (ii) characterise the pharmacokinetic profiles of cisplatin following intravenous and intratumoural administration. Collectively, this work seeks to demonstrate the utility of this translational model for preclinical lung cancer research and the evaluation of intratumoural drug delivery strategies.

## 2 Materials and methods

### 2.1 Experimental overview

The experiments conducted as part of this study consisted of both non-recovery procedures (**Figure 1A**; **Table 1**) and recovery procedures (**Figure 1B**; **Table 1**) in sheep. Using our previously published JSRV infection model (68, 27), sheep were infected with JSRV via localised bronchoscopic inoculation. CT scan data were collected at monthly intervals to track OPA development. The sheep included in this study were sourced from 3 cohorts of experimental JSRV infection studies. Across these cohorts, 54 sheep were experimentally infected with JSRV using a series of refined infection protocols. Twenty sheep developed gross OPA lesions suitable for translational research studies, while four additional sheep had only early lesions at euthanasia. Eight JSRV-infected sheep were included in the present study: five with gross OPA lesions and three that did not develop gross lesions.

**Figure 1:**
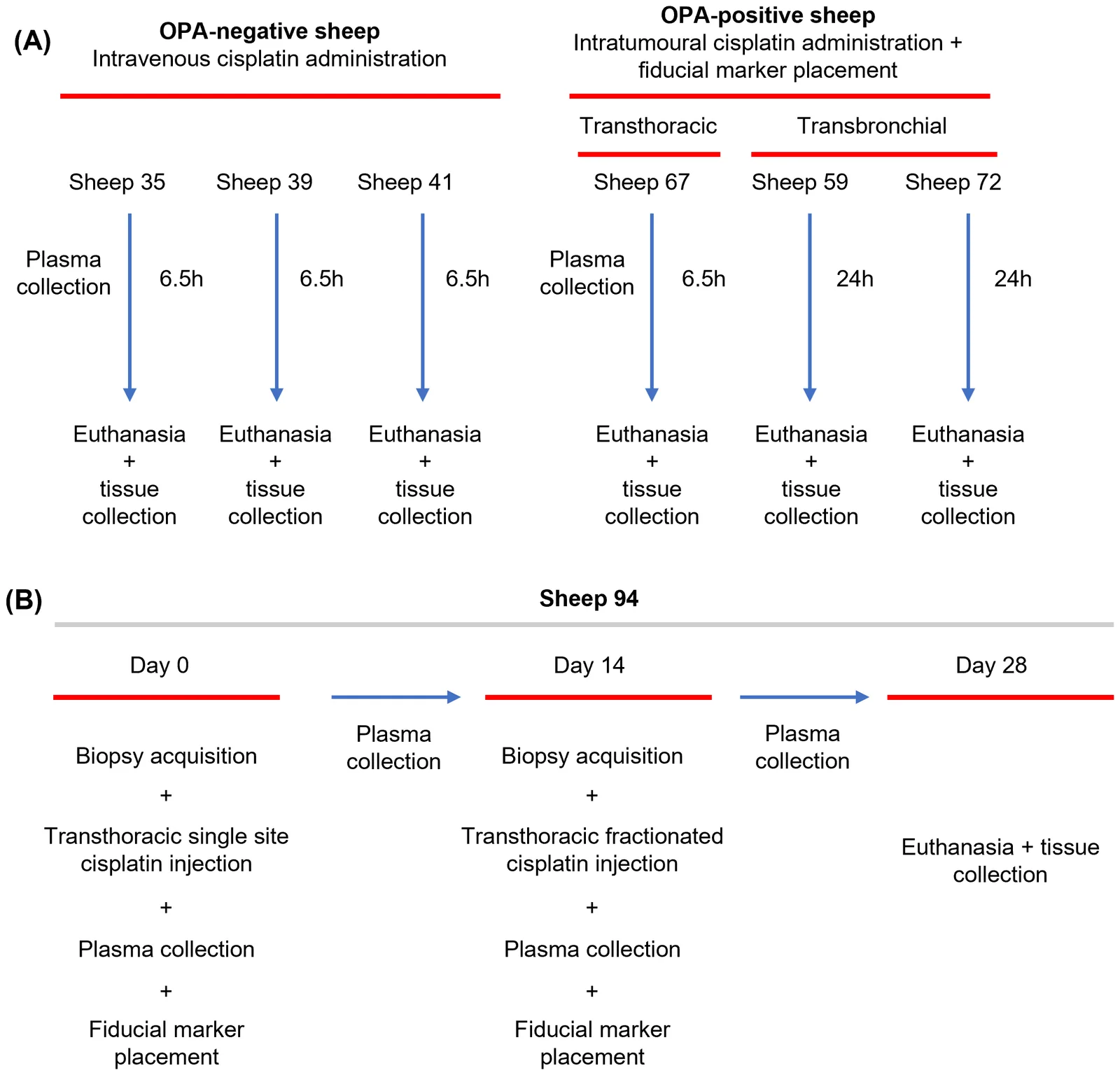
Experimental overview. Sheep were monitored longitudinally following JSRV inoculation and classified according to the presence or absence of OPA lesions at the time of study enrolment. **(A)** OPA-negative sheep were used to provide a reference for platinum distribution following intravenous cisplatin administration, whereas OPA-positive sheep underwent image-guided intratumoural drug delivery using either transthoracic or transbronchial approaches. Terminal pharmacokinetic studies included plasma sampling for either 6.5 h or 24 h following cisplatin administration prior to euthanasia and tissue collection. **(B)** One OPA-positive sheep (sheep 94) underwent a recovery study involving repeated CT imaging, tumour biopsies, and intratumoural cisplatin administration over a 28-day period. h, hours.

**Table 1:** Summary of animals, viral administration, lesion development, treatment groups, and sampling schedules included in the study.

Sheep	Virus titre	Virus volume	Lung lobe(s) targeted with virus	Number of gross lesions developed	Substance administered and delivery route	Recovery	Plasma sampling
35	Low	1 ml	RC	None	Cisplatin; IV	No	6.5 h
39	Low	1 ml	RC	None	Cisplatin; IV	No	6.5 h
41	Intermediate	1 ml	RC	None	Cisplatin; IV	No	6.5 h
67	Intermediate	1 ml	LC, RC, LVD3, RVD3	4	Cisplatin; transthoracic	No	6.5 h
59	Intermediate	1 ml	LC, RC, LVD3, RVD3	4	Cisplatin; transbronchial	No	24 h
72	Intermediate	1 ml	LC, RC, LVD3, RVD3	4	Cisplatin; transbronchial	No	24 h
94	Intermediate	1 ml (RC, RVD3); 40 ml (LC)	LC, RC, RVD3	5	Cisplatin and contrast agent; transthoracic	Yes	Days 0–28
85	Intermediate	1 ml (LC, RVD3); 40 ml (RC)	LC, RC, RVD3	4	Contrast agent; transthoracic	No	None

h, hours; IV, intravenous; LC, left cardiac; LVD3, left ventral diaphragmatic 3; RC, right cardiac; RVD3, right ventral diaphragmatic 3.

Three animals (sheep 35, 39 and 41) with no evidence of OPA development (assessed via imaging) underwent intravenous cisplatin administration in non-recovery procedures. The initial plasma sampling schedule of 6.5 h following intravenous cisplatin administration was designed to capture the early changes in plasma platinum concentrations, while permitting prolonged anaesthesia and serial blood sampling, with tissues acquired post-euthanasia for platinum analysis (**Figure 1A**). As experience with the protocol increased, plasma sampling was extended to 24 h for ITDD experiments.

Four sheep with evidence of OPA development (assessed via imaging) were chosen to undergo ITDD of cisplatin. Three animals (sheep 59, 67 and 72) underwent the intratumoural delivery of cisplatin (via transthoracic or transbronchial approaches) in non-recovery procedures, with fiducial markers placed at the site of cisplatin injection. These procedures were carried out 5, 2 and 4 months post-JSRV administration for sheep 59, 67 and 72 respectively. Platinum plasma levels were analysed up to 24 h post-administration, with tissues acquired post-euthanasia for platinum analysis (**Figure 1A**). In 1 sheep with evidence of OPA development (sheep 94, 8 months post-JSRV administration), intratumoural cisplatin injection experiments were carried out in a recovery protocol, comparing single site cisplatin injection versus a fractionated protocol, separated by a 2-week recovery time period. Biopsies taken at day 0 and day 14, before cisplatin injection, confirmed the presence of tumour at the injection sites. Fiducial markers were placed at the site of cisplatin injection. Plasma was collected over the course of the 28-day experiment to track platinum levels in the blood post-intratumoural injection. Tissues were acquired post-euthanasia for platinum analysis (**Figure 1B**).

A commercially available cisplatin formulation (1 mg/ml solution, Accord Healthcare) was used for both intravenous and intratumoural administration. No modifications were made to the formulation for the pharmacokinetic studies. In the recovery animal, cisplatin was mixed with contrast medium (Iohexol, Omnipaque 350 mg/ml, GE Healthcare) to permit CT visualisation of injectate distribution following intratumoural administration. One further sheep (sheep 85) was chosen for the transthoracic injection of contrast agent, which was carried out to test the procedure for targeting smaller tumour nodules in the sheep lung. This procedure was carried out in this sheep 5 months post-JSRV administration.

Results documenting the tracking of OPA development, tumour volumes and histopathology for sheep 59, 67 and 72 (used in the non-recovery ITDD experiments) have been previously published (68).

### 2.2 Animals

Studies were undertaken under a United Kingdom Home Office Project Licence in accordance with the Animals (Scientific Procedures) Act 1986 and with approval from the University of Edinburgh Animal Welfare and Ethical Review Body. Studies were designed and reported according to the “Planning Research and Experimental Procedures on Animals: Recommendations for Excellence” (36) and “Animal Research: Reporting of *In Vivo* Experiments” guidelines (37); these details are provided in our previous publications detailing the generation of the OPA model (68, 27). The study was also designed in accordance with the principles of Replacement, Reduction and Refinement (3Rs). Terminal procedures were initially used to establish and refine the ITDD techniques, optimise image-guided interventions and characterise platinum distribution before progressing to a single recovery animal to demonstrate the feasibility of longitudinal intratumoural therapy, tumour biopsy acquisition and CT monitoring of tumour response to treatment.

All lambs were obtained from the Moredun Research Institute’s high health status sheep flock and were Texel x Scotch Mule breed. They were vaccinated against Clostridia and Pasteurella and were weaned and housed together prior to transport to the University of Edinburgh’s Large Animal Research and Imaging Facility. Lambs were housed indoors on straw bedding in groups of at least 2 animals per pen with *ad libitum* access to food and water.

### 2.3 General anaesthesia

All bronchoscopic JSRV inoculation, intravenous and intratumoural cisplatin delivery procedures were conducted on anaesthetised animals. Although some aspects of anaesthesia varied between non-recovery and recovery procedures, the following protocol describes the general anaesthesia protocol used for these sheep. Any variations between non-recovery and recovery procedures are highlighted. Drugs used, including names, manufacturers, doses and routes of administration are provided in **Supplementary Tables 1–4**.

Concentrate feed was withheld on the morning of anaesthesia, but access to hay and water was permitted until pre-anaesthetic medication was administered via a pre-placed jugular venous cannula (Intraflon 16 g x 52 mm, Vygon). When sedation was present (after approximately 5 min), anaesthesia was induced with intravenous propofol, given until conditions for endotracheal intubation (with a cuffed tube, internal diameter 7.5-10.0 mm) were present. Anaesthesia was maintained using a combination of partial intravenous anaesthesia and isoflurane vaporised in oxygen and medical air (FiO_2_ approximately 0.5), delivered using ‘circle’ breathing systems from anaesthesia workstations with integral ventilators (Aestiva/5, GE Datex-Ohmeda). Viral filters (Inter-Guard breathing filter, Intersurgical Ltd.) were positioned between the endotracheal tube and breathing system. Volume-controlled positive pressure ventilation was implemented to achieve tidal volumes of 8–10 mL/kg and end-tidal CO_2_ partial pressures approximating 5.3 kPa. In female sheep, a urinary catheter was placed per vaginum to allow collection of urine. A cannula (Biovalve Safe 20 g x 32 mm, Vygon) was placed in the auricular artery to allow for invasive blood pressure measurement and arterial blood sampling. During anaesthesia, pulse rate, pulse oximetry, temperature, capnography, spirometry, and inspired and expired gases (O_2_, CO_2_ and inhalant anaesthetic agent) were monitored (S/5 Compact Anaesthesia Monitor, GE Datex-Ohmeda). Additional supportive therapies were administered as required (**Supplementary Tables 1–4**). If a pneumothorax occurred, this was managed with intermittent thoracocentesis using a percutaneously placed 19 g hypodermic needle placed under CT guidance.

In the recovery procedures, ventilation was supported manually until spontaneous breathing of room air achieved an SpO_2_ > 90%. The trachea was extubated once active chewing was present, and the sheep were monitored continuously thereafter until they could stand unassisted. An intravenous jugular catheter was left in place after recovery to allow repeated blood sampling.

### 2.4 Bronchoscopy and JSRV administration

JSRV production and administration was performed as previously described (68, 27). See **Supplementary Table 5** for information regarding the sex, age, and weight of sheep at the time of JSRV administration. Briefly, bronchoscopy was performed using a 6 mm x 1000 mm slim video Gastroscope (VET-6010HD, Ves Custom Optics) to inoculate virus into distinct lung lobes (**Table 1**). Virus aliquots (diluted in PBS) were produced as previously described and remained frozen on dry ice until immediately prior to use, when they were defrosted on wet ice. Either low or intermediate doses of virus were used (**Table 1**), which were equivalent to 80 and 400 ng HIV RT, respectively, as assessed by the RT activity assay. For catheter airway delivery, the bronchoscope was introduced via the endotracheal tube and positioned within the segmental bronchus of the cardiac lung lobes. A sterile instillation catheter (Ves Custom Optics) was introduced down the working channel of the bronchoscope. The viral dose was drawn up into a luer lock syringe, attached to the instillation catheter for delivery and delivered into subsegmental bronchi leading from the segmental bronchus. For intra-parenchymal delivery into the diaphragmatic lobes, the bronchoscope was positioned within the segmental bronchus of the ventral diaphragmatic lung lobe 3. Using a 23 g Monarch aspirating biopsy needle (Auris Health), passed down the working channel of the bronchoscope, the virus was drawn up into a luer lock syringe and attached to the injection device for delivery. The needle was advanced through a lower airway wall to deliver the virus into lung parenchyma.

### 2.5 CT scanning and image analysis

A multi-slice SOMATOM Definition AS 64 slice fan beam CT machine (Siemens Healthcare Ltd.) was used to obtain thoracic CT scans from sheep positioned in sternal recumbency, as previously described (68, 27). CT scans were acquired using the following parameters: 120 kVp, 73 mA, 1.2 pitch, 76.59 mm/s table speed, 23 mm/rotation table feed, 512 × 512 matrix size, and 2 mm slice thickness. A continuous positive airway pressure protocol was applied to induce apnoea and standardise lung volume for CT acquisition. Reconstructions were performed using the Siemens convolution kernel I70f2, SAFIRE strength 2. CT calibration with a phantom was performed on a weekly basis. Baseline (immediately prior to JSRV administration) and then monthly serial thoracic CT scans were acquired to assess the nature and extent of OPA tumour progression. CT scans were assessed for the presence of normal and abnormal lung patterns, as previously described (68, 27). An imaging-based diagnosis of OPA was made on the presence of hyperattenuating lung parenchyma in the lobes that were inoculated with JSRV. This hyperattenuation was tracked over progressive CT scans and confirmed to increase in volume.

Digital Imaging and Communications in Medicine data were imported into Analyze 12.0 software (AnalyzeDirect, Overland Park) for quantitative analysis as previously described (68, 27). Briefly, all parenchymal tissue judged to be abnormal and consistent with an OPA lesion (hyperattenuation and the presence of abnormal lung and vascular patterns) was segmented into a region of interest. Segmentation was performed on 2D CT slices using axial, coronal and sagittal views to generate 3D volumes. Region of interest delineation began with manually placing a ‘seed’ pixel, followed by expanding the intensity threshold to define the structure’s boundary. Manual boundaries were drawn to prevent the seeded region from growing into neighbouring structures with similar pixel intensities. Regions of interest were propagated across adjacent slices and manually adjusted to follow structure boundaries. Regions of interest were refined through visual inspection in 2D and 3D views to finalise the 3D volumes.

### 2.6 Intravenous cisplatin administration

Three sheep (sheep 35, 39 and 41), which did not show any imaging changes consistent with OPA development 10 months post virus administration, were selected to undergo an intravenous cisplatin pharmacokinetic protocol. For these systemic delivery experiments, an estimation of body surface area was calculated using the equation: 0.09 × W^0.67^ (where W = weight in kg) (38). A clinically relevant dose of 80 mg cisplatin/m^2^ body surface area was used for all 3 sheep (doses ranged from 118–124 mg cisplatin per sheep). Animals were anaesthetised as previously described and positioned in sternal recumbency. Catheters were placed in both left and right jugular veins, one was used for cisplatin delivery and the other for obtaining blood samples. Cisplatin was diluted with 500 mL NaCl 0.9% and administered over 30 min using an infusion pump. Blood samples were collected into ethylenediaminetetraacetic acid (EDTA) tubes at specific time points, ranging from directly before cisplatin injection to 6.5 h post-injection (**Supplementary Table 6**), and placed onto ice. Plasma was recovered by centrifugation for 15 min at 2,000 x g at 4 °C and was stored at -80 °C post-acquisition.

### 2.7 Transthoracic cisplatin administration

Two sheep that showed imaging changes consistent with OPA development (sheep 67, non-recovery procedure carried out 2 months post virus administration; sheep 94, recovery procedure carried out 8 months post virus administration) were anaesthetised as previously described and positioned in sternal recumbency. In both the recovery and non-recovery procedures, the thorax was clipped and aseptically prepared between the caudal border of the last rib and the caudal border of the scapula. The dorso-ventral margins extended from the dorsal spinous process of the thoracic vertebrae ventrally to the sternum. The area was then four quarter draped for surgery. An initial CT scan was taken to assess intrathoracic pathology and identify the OPA lesion site for ITDD. Injection sites were planned to target the centre of the OPA lesion, with one lesion per sheep selected prospectively based on technical suitability for image-guided injection. Lesions were selected so the needle path would avoid bullae, fissures, visible blood vessels and large bronchioles. Once the site was identified, the CT slice number of the axial plane was obtained and lasers on the bore were used to identify this site on the thorax, which was then marked with a sterile pen. A 21 g marker needle was placed in the skin and a CT scan was used to confirm position. The distance between the skin and pleura was measured at the anticipated penetration site. The marker needle was removed and replaced with the injection needle and stylet (Gangi-HydroGuard 17 g, Apriomed). The needle was advanced through the chest wall (based on the pre-measured distance from the initial CT scan). A CT scan was taken to verify correct needle position and angulation. If necessary, the needle could be repositioned without risk of lung damage as the needle had not yet penetrated the visceral or parietal pleura. Once in the correct position, under breath hold conditions, the needle was advanced through the parietal and visceral pleura and into OPA tissue, with mechanical ventilation resumed thereafter; repeat CT scans were taken following each needle advancement (made under breath hold conditions) and measurements were made determining the distance from needle tip to the point of desired ITDD.

In the non-recovery sheep experiment (sheep 67), following placement of the needle tip centrally within OPA tissue, the stylet was removed and 2 mg of cisplatin was injected slowly over 2 min, with the total 2 ml volume delivered at a single injection site. In the recovery sheep experiment (sheep 94), the injection volume of cisplatin was 25% of the left cardiac (LC) tumour volume (calculated through reconstructions made using CT scan images taken on the day of injection); 26.5 mg of cisplatin (26.5 ml), with 20% volume contrast added to enable visualisation of cisplatin localisation post-injection (Iohexol, Omnipaque 350 mg/ml, GE Healthcare), was given at 1 ml per min using a syringe driver. For the day 0 injection, the total volume was delivered at a single injection site, whereas for the day 14 injection, the total volume was equally divided into 3 fractions.

Following the cisplatin injections, in both the non-recovery and recovery procedures, under breath hold conditions, a 4 mm x 4 mm fibered platinum coil fiducial marker (VortX, Boston Scientific) was delivered into the OPA tissue through the needle to mark the injection site. The needle was then removed. CT scans were performed to evaluate fiducial marker positioning, assess any immediate post-operative complications such as pneumothorax or haemorrhage, and also to evaluate contrast deposition (recovery experiment). Blood samples were collected into EDTA tubes at specific time points, from directly before cisplatin injection up to 6.5 h post-injection in the non-recovery sheep (**Supplementary Table 7**), and from directly before cisplatin injection up to 2 weeks post-injection in the recovery sheep experiment (**Supplementary Table 8**) and placed on ice. Plasma was recovered by centrifugation for 15 min at 2,000 x g at 4 °C and was stored at -80 °C post-acquisition.

Sheep 85 was used to develop an ITDD technique for targeting smaller tumour nodules that arose in the diaphragmatic lobes after intra-parenchymal JSRV injection. The tumour that developed in the right ventral diaphragmatic 3 lobe was targeted using the same procedure as outlined above. In this experiment, under breath hold conditions, 2 ml of contrast agent (Iohexol, Omnipaque 350 mg I/ml, GE Healthcare) was injected at 1 ml per min using a syringe driver, with the total volume delivered at a single injection site. The needle was then removed, with CT scans taken to assess for any immediate post-operative complications such as pneumothorax or haemorrhage, and also to evaluate contrast deposition.

### 2.8 Transbronchial cisplatin administration

Two sheep that showed imaging changes consistent with OPA development were anaesthetised as previously described and positioned in sternal recumbency (sheep 59, non-recovery procedure carried out 5 months post virus administration; sheep 72, non-recovery procedure carried out 4 months post virus administration). An initial CT scan was taken to assess intrathoracic pathology and identify OPA lesions for ITDD. Bronchoscopy was performed using a 6 mm x 1000 mm slim video Gastroscope (VET-6010HD, Ves Custom Optics). The bronchoscope was introduced via the endotracheal tube and positioned within the segmental bronchus of the lung lobe containing the OPA tumour. A 23 g Monarch aspirating biopsy needle (Auris Health) was passed down the working channel of the bronchoscope. A CT scan was taken to ensure correct placement of the scope; the needle was then extended from the sheath and advanced, under breath hold conditions, 1.5 cm into lung parenchyma. 2 mg cisplatin (2 ml) was injected slowly over 2 min. The needle was retracted. Following the injection, a 4 mm x 4 mm fibered platinum coil fiducial marker (VortX, Boston Scientific) was delivered into the airway lumen, directly adjacent to the injection site, before the scope was removed. Video recordings were captured as mp4 files. CT scans were performed to evaluate fiducial marker positioning and to assess any immediate post-operative complications such as pneumothorax or haemorrhage. Blood samples were collected into EDTA tubes at specific time points, from directly before cisplatin injection up to 24 h post-injection (**Supplementary Table 9**) and placed on ice. Plasma was recovered by centrifugation for 15 min at 2,000 x g at 4 °C and was stored at -80 °C post-acquisition.

### 2.9 Biopsy procedure

Sheep 94 underwent transthoracic biopsy on days 0 and 14, immediately prior to cisplatin injection. Following transthoracic injection needle placement at the site of intended injection, under breath hold conditions, a Tru-Cut 18 g biopsy needle (Mission Disposable Core Biopsy Instrument, Becton Dickinson) was introduced and used to obtain 2 x 10 mm tissues sections. Tissue samples for histological analysis were placed in 4% formaldehyde (Genta Medical) for at least 24 h.

### 2.10 Post mortem examination

Animals were euthanised at the end of the procedures for post mortem examination. Euthanasia was performed using intravenous sodium pentobarbital (Pentoject, Animalcare) at 80 mg/kg. The trachea, lungs, and caudal tracheobronchial lymph nodes were removed from the thoracic cavity. Post mortem evaluation of gross specimens was performed, which included photographic documentation of the lungs and any associated pathology. The injected OPA tumour was excised and underwent fluoroscopic imaging to identify the fiducial marker. This allowed tissue sampling from the injected lobe at the injection site, and also at sites 2 and 4 cm distant from it. Approximately 200 mg tissue samples were obtained in triplicate from tumour areas, lung and tracheobronchial lymph nodes. Samples were snap frozen on dry ice and then stored at -80 °C for subsequent platinum analysis. Tissue samples for histological analysis were taken from the same areas in the injected lobe and from any other lobes which had gross lesions. These samples were placed in 4% formaldehyde (Genta Medical) for at least 24 h.

### 2.11 Histopathology

Following fixation, tissue samples underwent routine processing through graded alcohols prior to embedding in paraffin wax. Four-micron serial sections were cut and stained with haematoxylin and eosin (H&E). Whole slides were scanned using a Hamamatsu NanoZoomer (Hamamatsu Photonics, Shizuoka, Japan). Slides were assessed by a board-certified veterinary pathologist.

### 2.12 Serum and tissue platinum analysis

All chemicals used for sample pre-treatment and mass spectrometry were of analysis grade or of high purity. HNO_3_ of trace metal analysis grade was purchased from ThermoFisher Scientific and de-ionised water 18.2 MΩ was produced using an Arium II system (Sartorious). The calibration standard solution of platinum, as well as a solution of the element iridium (Ir) used as internal standard, were prepared using single element stock solutions with a concentration of 1000 mg/mL (both PlasmaCAL grade, SPE Science). For quality control aspects on the relevant ultra-trace level, a reference sample SEROnorm™ Trace Elements in Whole blood L-1 (AB Scientific) was included in the analytical procedure. Although the platinum concentrations were not certified in these samples, they were used as reference matrices for additional control experiments.

For the analysis of platinum levels in tissue, 176 ± 38 mg of lung, lymph node or OPA tumour was digested in 9 times its volume in concentrated HNO_3_ at 80 °C in disposable soda glass tubes for 1 h on a block heater type BT5D (Grant Instruments). The volume of tissue was presumed to be 1 g/ml, so for example, a 100 mg tissue sample would have been digested in 900 µl HNO_3_. After digestion was completed, all samples were diluted by a factor of five with ultrapure water to get a final digestion solution of approximately 20% HNO_3_. For each digestion batch, 3 reagent blanks were carried through the same procedure as the samples. Prior to analysis, the samples were further diluted by a factor of 10 with de-ionised water to reduce the acid strength to <2% v/v. The plasma samples were digested by adding 25 or 50 µl of the plasma to 25 or 50 µl of concentrated HNO_3,_ shaken for a few seconds until the solution turned yellow/brown, then diluted with de-ionised water to 4 ml. After allowing any undigested plasma material to settle, a 1 ml aliquot was taken out for analysis. Samples were prepared in triplicate, except for sheep 72, which was repeated in quadruplicate due to the lower concentrations of platinum detected.

All samples were analysed for platinum content using a single quadrupole inductively coupled plasma mass spectrometer (ICP-MS; type 7900, Agilent). The key instrumental setup and method settings of the ICP-MS were: plasma, auxiliary and nebuliser gas flows of 15, 0.9 and 1.09 L/min respectively, RF power 1550 W and the spray chamber cooled to 2 °C. Helium gas mode in the collision cell was used to improve background noise; the Helium flow rate was 4.3 mL/min. An external calibration curve for platinum of the interference-free isotope ^195^Platinum was performed over a range of 0.001-10 µg/L at the start of the analysis. On-line addition and correction with an internal standard (200 µg/L iridium) with the measured isotope ^193^Ir and the correction for the chemical blank were also applied. For the method validation, the average methodological limit of detection, limit of quantification, and the blank were calculated as 0.008, 0.023 and 0.004 µg/L respectively, and were all an order of magnitude lower than the lowest samples analysed. An initial set of analysis was carried out to assess platinum washout and spike recovery from a similar blood matrix (SEROnorm™ Trace Elements in Whole blood L-1). The concentration of platinum is given a “< value” on the certificate, therefore the SEROnorm™ samples were used to check for blanks and also for the determination of the trueness by spike recovery at concentrations of 0.01, 0.1 and 1 µg/L platinum. Spike recoveries carried out across the data sets had an average recovery of 103.5 ± 5.6%. The variation within and between analysis batches were determined by duplicate and triplicate measurements of selected samples.

## 3 Results

### 3.1 Intravenous cisplatin administration shows rapid plasma platinum peak with fast decline

Three sheep (35, 39 and 41) that did not show any imaging changes consistent with OPA gross lesion development (**Figures 2A, B**) were selected to undergo an intravenous cisplatin pharmacokinetic protocol. Plasma was collected directly before cisplatin administration, and at specific time points up to 6.5 h post-administration. A rapid increase in plasma platinum levels was observed during the 30 min infusion in each sheep, which peaked between 5.25-6.53 mg/L at the end of the injection. This was followed by a rapid decline in plasma platinum concentrations, which subsequently approached a relatively stable plateau (1 mg/L) by approximately 180 min post-infusion. This level was maintained until completion of the study at 6.5 h (**Figure 2C**).

**Figure 2:**
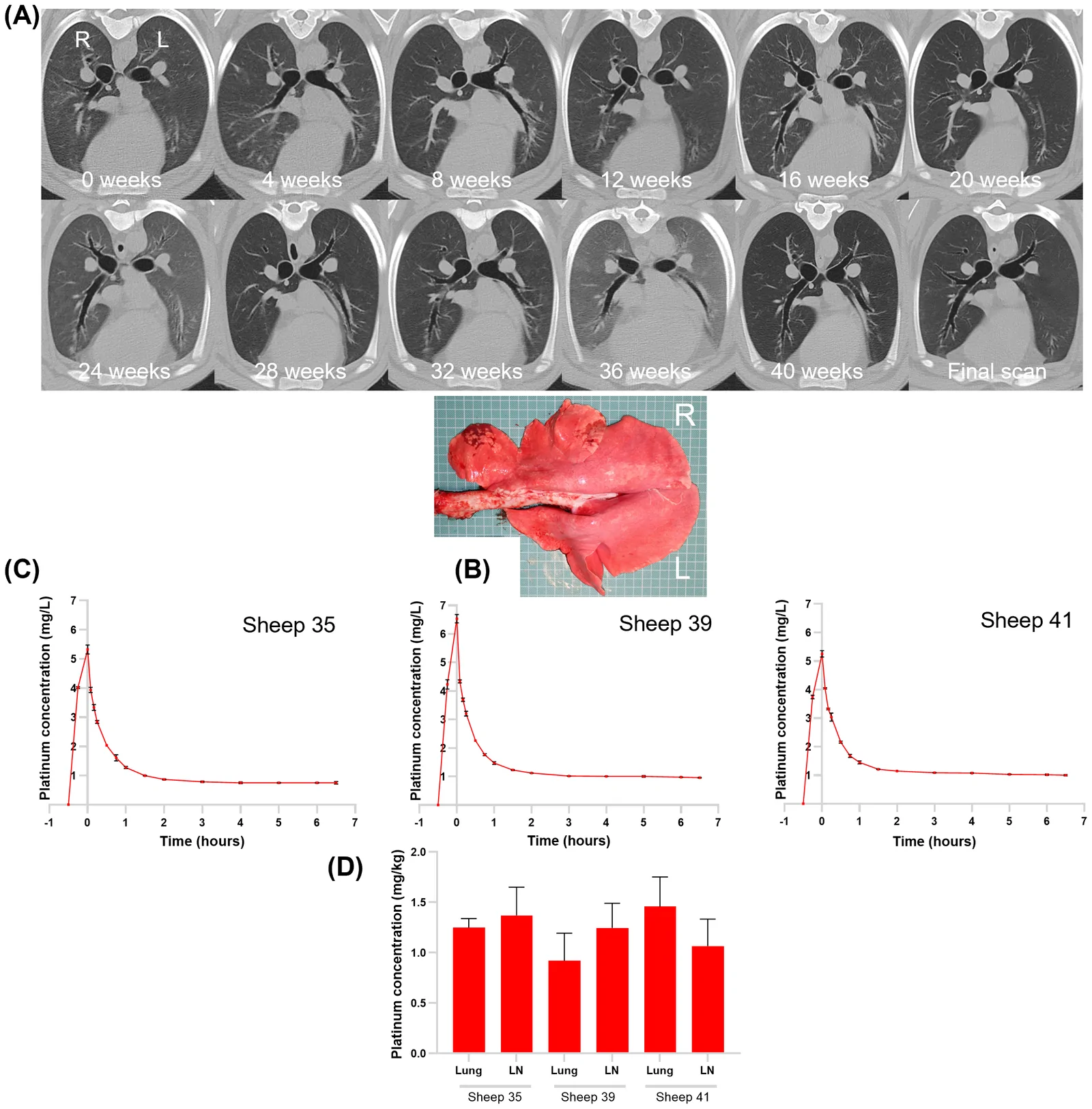
Plasma and tissue platinum concentrations following intravenous cisplatin administration. Representative CT and gross lung images are shown to document the absence of imaging and gross pathological evidence of OPA in the sheep used for intravenous cisplatin administration. **(A)** Serial axial CT images highlighting the left and right cardiac lung lobes in a JSRV-infected sheep with no gross tumour formation (sheep 41). These images demonstrate normal appearing lung seen at all time points, characterised by hypoattenuating lung parenchyma, through which air-filled (black) bronchi and bronchioles run. L, left; R, right. **(B)** Gross post mortem photograph of the dorsal aspect of the ovine lungs with no evidence of gross lesion development. L, left; R, right. **(C)** Three sheep that did not show any imaging changes consistent with OPA development were selected to undergo an intravenous cisplatin pharmacokinetic protocol. Plasma platinum concentrations were analysed directly pre-infusion and for 6.5 h post-infusion, with cisplatin injection starting at -0.5 h and lasting until 0 h (mean ± standard deviation; technical replicates = 2-3). **(D)** Tissue platinum concentrations were analysed from samples acquired at post mortem (mean ± standard deviation; technical replicates = 3; LN, lymph node).

The levels of platinum within lung and tracheobronchial lymph node tissue were also assessed to provide a reference for tissue platinum distribution following intravenous cisplatin administration. Platinum levels were similar in the lung and lymph node tissue between each of the sheep, ranging between 0.92-1.46 mg/kg (**Figure 2D**).

### 3.2 Tracking OPA development in cardiac and diaphragmatic lobes through CT scanning

Serial monthly CT scans taken over the course of the study were assessed for the presence of normal and abnormal lung patterns. Advanced lesions typically presented as large hyperattenuating areas that cause small airways to collapse (**Figure 3A**). Sheep exhibiting areas of hyperattenuating lung in the instilled/injected lobes were given a preliminary CT-based diagnosis of OPA. Three dimensional reconstructions of the tumours were produced using the monthly CT scans, which enabled close tracking of tumour size over time (**Figure 3B**). These 3D reconstructions were also used to calculate the volumes of the tumours targeted in the ITDD experiments (**Table 2**).

**Figure 3:**
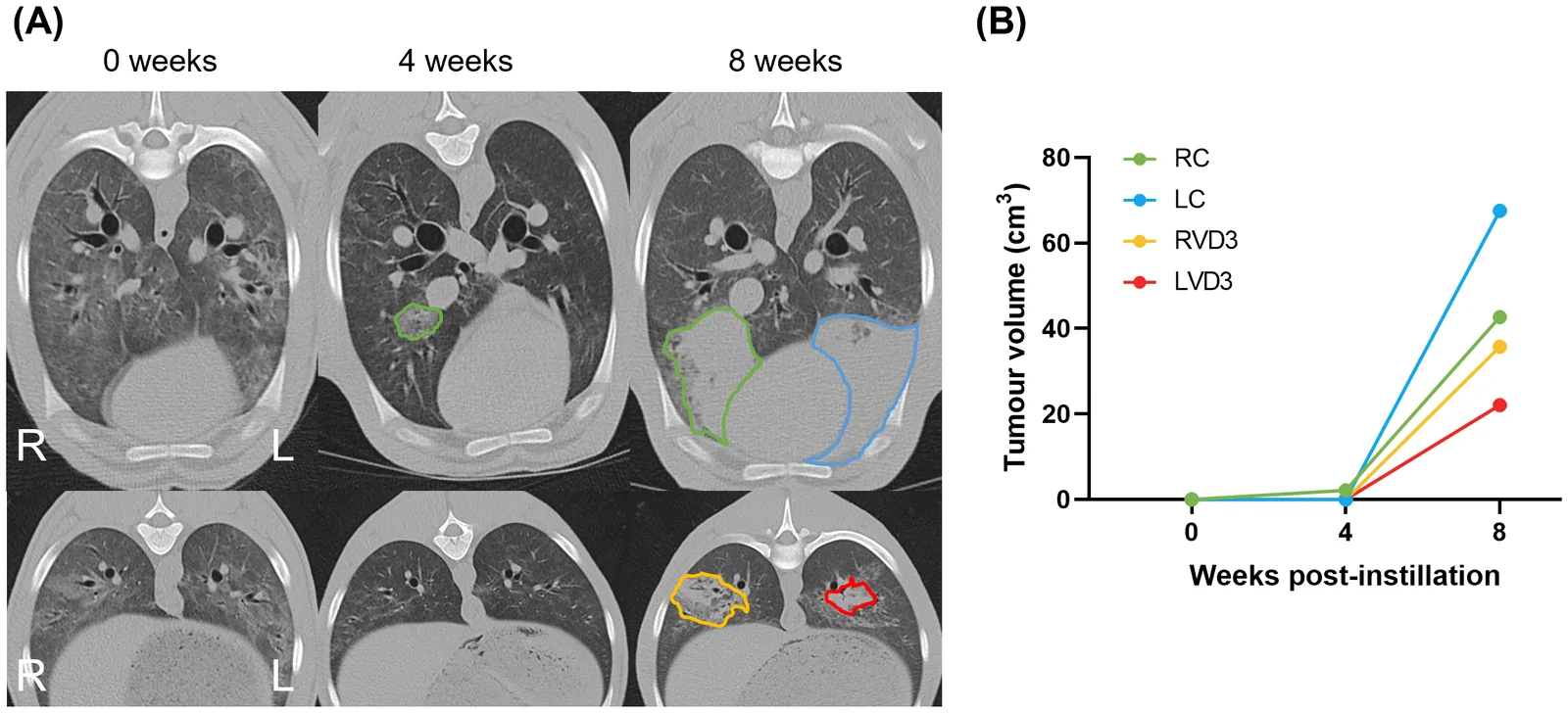
Serial CT and tumour growth rates documenting OPA tumour development at the 4 JSRV inoculation sites. **(A)** Serial axial CT images highlighting the left and right cardiac lung lobes (top panel) and the left and right VD3 lobes (bottom panel) for sheep 67. CT scans indicated OPA development in the right cardiac lobe 4 weeks post-instillation. Large tumours were evident in both of the catheter instilled lobes (LC, blue; RC, green) and both of the needle injection site lobes (LVD3, red; RVD3, yellow) 8 weeks after JSRV administration. L, left; R, right. **(B)** Graph of tumour volumes from all 4 JSRV inoculation sites in sheep 67. These tumour volumes have previously been reported (paper under review by Veterinary Pathology, reference VET-26-FLM-0077). RC, right cardiac; LC, left cardiac; RVD3, right ventral diaphragmatic 3 lobe; LVD3, left ventral diaphragmatic 3 lobe.

**Table 2:** OPA tumour volumes targeted in the intratumoural drug delivery and contrast injection experiments.

Sheep	Tumour targeted	Size (cm^3^)
67 (cisplatin, transthoracic injection, non-recovery procedure)	Left cardiac	67.57
59 (cisplatin, transbronchial injection, non-recovery procedure)	Left cardiac	222.30
72 (cisplatin, transbronchial injection, non-recovery procedure)	Right cardiac	113.29
94 (cisplatin + contrast agent, transthoracic injection, recovery procedure)	Left cardiac (day 0)	130.40
	Left cardiac (day 14)	208.67
85 (contrast agent, transthoracic injection, non-recovery procedure)	Right ventral diaphragmatic 3	22.88

### 3.3 Transbronchial and transthoracic cisplatin injection in non-recovery sheep

Three sheep (59, 67 and 72) with gross OPA tumours were chosen to develop protocols for the ITDD of cisplatin. Transbronchial intratumoural injections were carried out for sheep 59 and 72 (**Figure 4A**; see **Supplementary Figure 1** for the CT scans of sheep 59), with transthoracic intratumoural injection performed for sheep 67 (**Figure 4B**). The whole procedures were performed under terminal anaesthesia. Fiducial markers were placed at the injection site for each sheep (**Figure 4A, B**). Cisplatin injection was successfully performed in all sheep without evidence of major procedure-related complications, including haemorrhage, acute systemic adverse reactions or acute post-procedural deterioration. In one animal (sheep 67), a moderate pneumothorax developed following needle withdrawal; this was managed intraoperatively with intermittent thoracocentesis and did not result in further adverse events. After euthanasia, the lungs were removed and photographed (**Figure 4C**), and the injected lobes were imaged with fluoroscopy to identify the fiducial marker and point of injection (**Figure 4D**). Confirmation that the injection site had been found and sampled accurately was done through identification of the needle tract on H&E-stained sections (**Figure 4E**).

**Figure 4:**
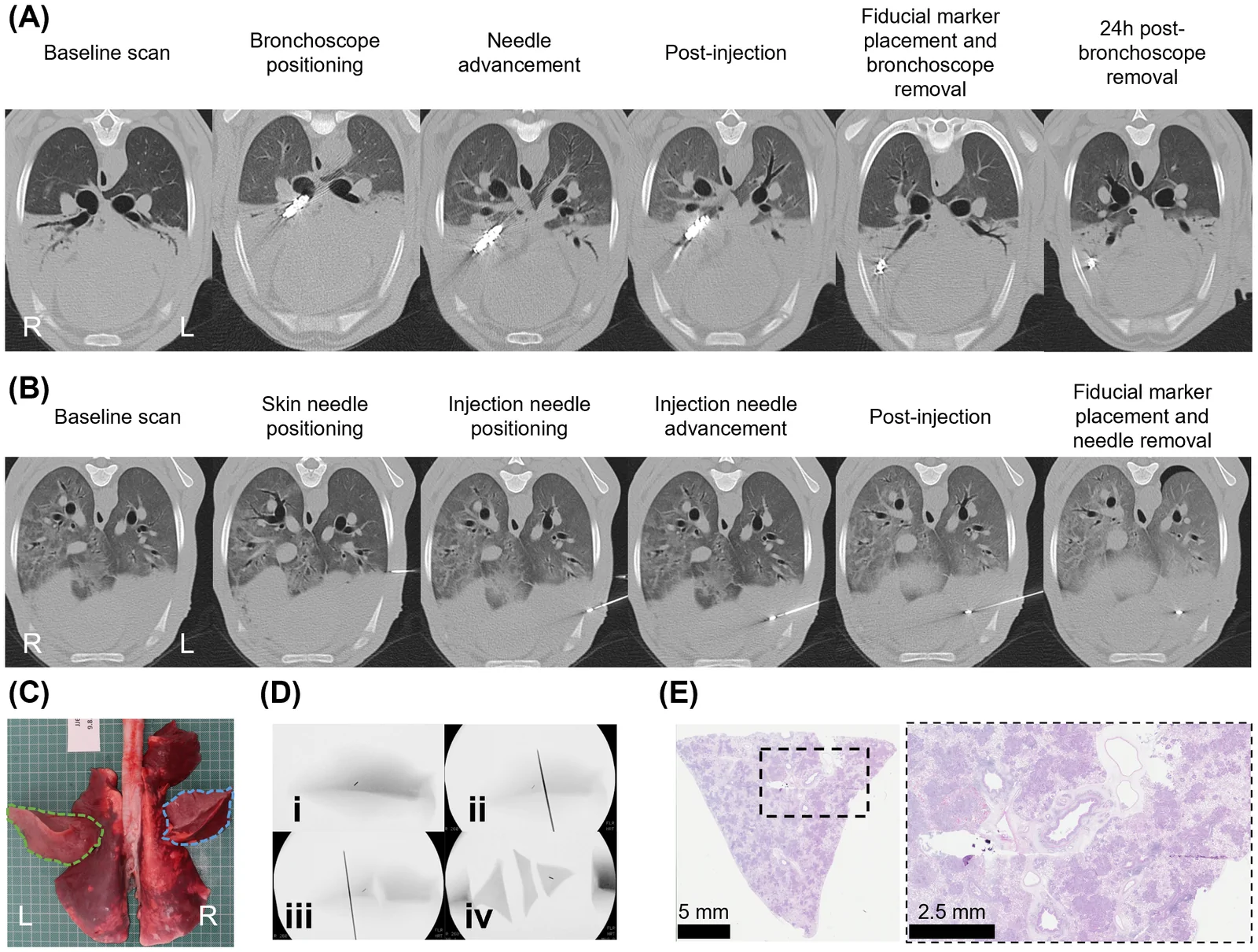
Transbronchial and transthoracic cisplatin injection techniques. **(A)** Axial CT images showing the transbronchial injection of cisplatin into the right cardiac lobe tumour of sheep 72. L, left; R, right. **(B–E)** Axial CT images, post mortem, fluoroscopy and histology showing the complete intratumoural drug delivery protocol of sheep 67. **(B)** Axial CT scans taken during the transthoracic injection of cisplatin into the left cardiac lobe tumour. A small pneumothorax occurred following needle retraction. L, left; R, right. **(C)** Gross post mortem photograph of the dorsal aspect of the ovine lungs, with evidence of gross lesion development in the left (green dashes) and right (blue dashes) cardiac lobes. L, left; R, right. **(D)** Ex vivo fluoroscopic imaging of the cisplatin-injected lung lobe. **(D i)** Platinum fiducial marker identified in the central area of the intact left cardiac lobe. **(D ii, iii)** Under live fluoroscopic imaging, a scalpel blade is used to cut either side of the fiducial marker to isolate the injected area. **(D iv)** Isolated tumour area contains the fiducial marker. **(E)** H&E-stained section of the left cardiac lobe OPA lesion containing the injection site. Tissue shows replacement of normal alveolar architecture by a well-differentiated epithelial neoplasm exhibiting predominantly acinar and papillary growth patterns, consistent with OPA.

To analyse the release of cisplatin into the bloodstream post-intratumoural injection, plasma levels of platinum were recorded before cisplatin injection and up to the point of euthanasia (**Figure 5**). The first sheep used in these experiments (sheep 67, transthoracic injection) was maintained under anaesthesia for 6.5 h post-injection, with the subsequent sheep anaesthetised and plasma collected for 24 h post-injection. In sheep 67, a peak in plasma platinum levels of 0.047 mg/L was observed 2 h post-injection; by the end of the blood sampling protocol (6.5 h post-injection), the levels had dropped to 0.021 mg/L. For sheep 59 (transbronchial injection), plasma platinum levels peaked at 0.050 mg/L at 5 min post-cisplatin injection, with the plasma levels of platinum decreasing rapidly after this and stabilising 2 h post-injection at approximately 0.012 mg/L; the concentration of platinum in the blood was 0.011 mg/L by the end of the blood sampling protocol (24 h post-injection). In sheep 72 (transbronchial injection), a peak in plasma platinum levels of 0.017 mg/L was observed 30 min post-injection; 24 h post-injection, the levels had dropped to 0.011 mg/L.

**Figure 5:**
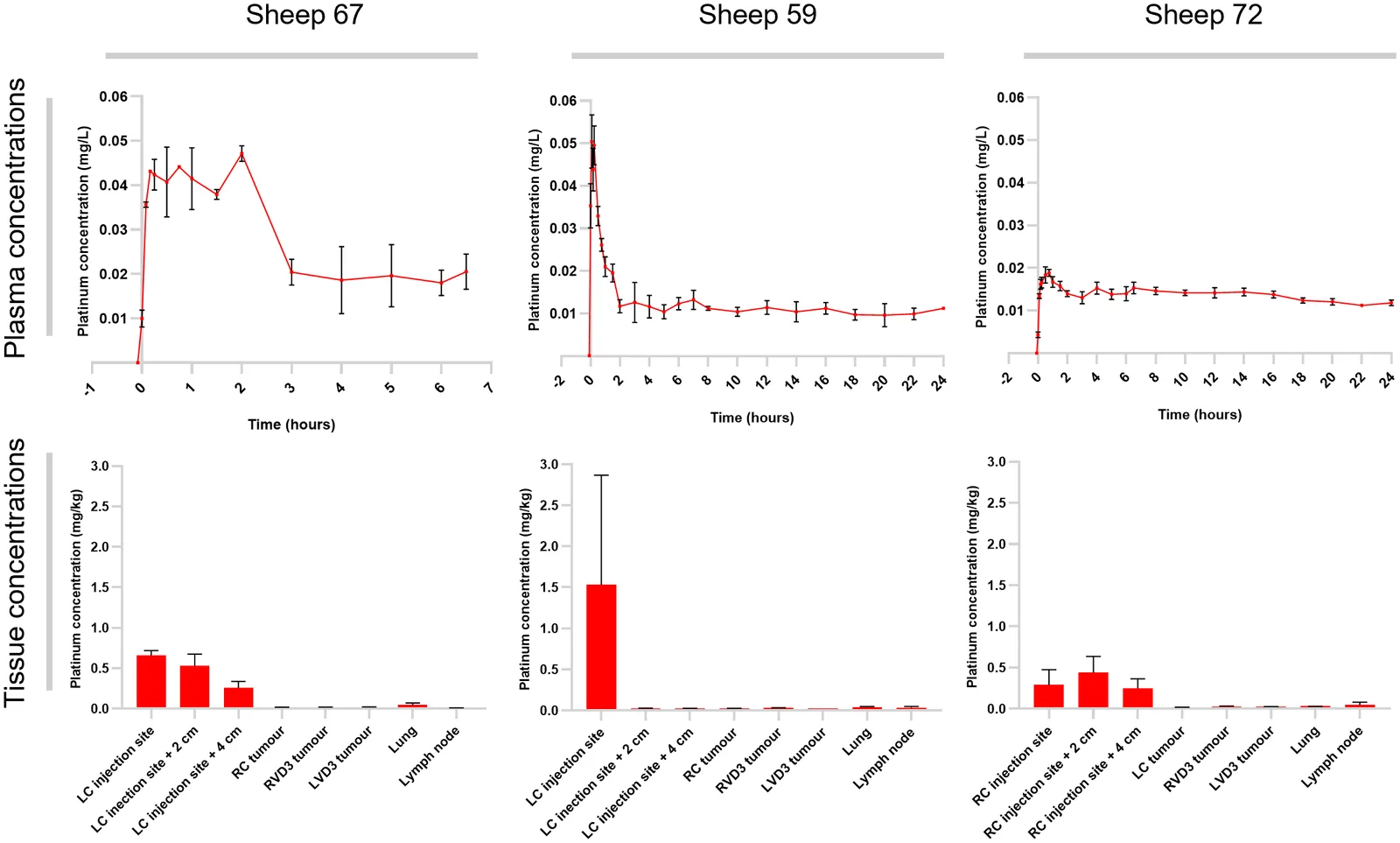
Plasma and tissue platinum levels post-intratumoural cisplatin injection. Cisplatin was injected intratumourally into 3 sheep that developed gross OPA lesions. Transthoracic intralesional injection of cisplatin was performed for sheep 67, with transbronchial intralesional injections carried out for sheep 59 and 72. Plasma levels of platinum were recorded both before cisplatin injection, and up to 24 h post-injection. The level of platinum in tissue acquired at post mortem was also analysed for each of the sheep. Tissues analysed included the tumour injection site, tumour tissue at distances 2 cm and 4 cm away from this site, non-injected tumour tissue, normal lung tissue and lymph node tissue (mean ± standard deviation; technical replicates = 2-4. LC, left cardiac; RC, right cardiac; RVD3, right ventral diaphragmatic 3; LVD3, left ventral diaphragmatic 3).

Following post mortem examination, tissues were also taken to assess platinum concentrations in the injected tumour (at the injection site and in tissues at 2 cm intervals from the injection site), in tumours present in other lobes, and also in normal lung and local lymph node tissue (**Figure 5**). Platinum concentrations were highest at the tumour injection site in all three sheep (approximately 1.53, 0.66 and 0.29 mg/kg for sheep 59, 67 and 72, respectively), with substantially lower concentrations detected in non-targeted tumour tissue, normal lung and tracheobronchial lymph nodes (**Figure 5**). These findings demonstrate preferential deposition of platinum within the targeted tumour following intratumoural administration. The presence of OPA at the tumour injection sites, and at the other JSRV instillation/injection sites, was confirmed through histological analysis.

### 3.4 Transthoracic cisplatin injection in a recovery sheep experiment

Intratumoural cisplatin injection experiments were carried out in a recovery protocol, comparing single site cisplatin injection versus a fractionated protocol, separated by a 2-week recovery time period. This experiment was performed to determine the feasibility of repeated intratumoural injections, tumour biopsies and recovery anaesthesia using our OPA model. The cisplatin was mixed with contrast agent to visualise drug distribution within the tumour on post-injection CT scans. The single site cisplatin injection was carried out on day 0 into the LC lobe (**Figure 6A**). However, much of the cisplatin was observed in the left ventral diaphragmatic 1 (LVD1) tumour on post-injection CT scans; this tumour most likely developed in this lobe after spread of the virus from the initial inoculation sites. The fractionated protocol, which involved 3 transthoracic intratumoural cisplatin injections in different regions of the LC tumour, was performed 14 days later (**Figure 6B**), with the cisplatin observed to remain within the LC tumour, as observed through the post-injection CT scan. Both the single site and fractionated protocols were carried out without any issues, and the sheep recovered uneventfully from anaesthesia. Biopsies taken at day 0 and day 14, before cisplatin injection, confirmed the presence of tumour at the injection sites and were carried out without complications (**Figures 6A, B**). Euthanasia was carried out 28 days after the first cisplatin injection. Fiducial markers placed at the time of cisplatin injection had not deviated from their initial positions (**Figure 6C**).

**Figure 6:**
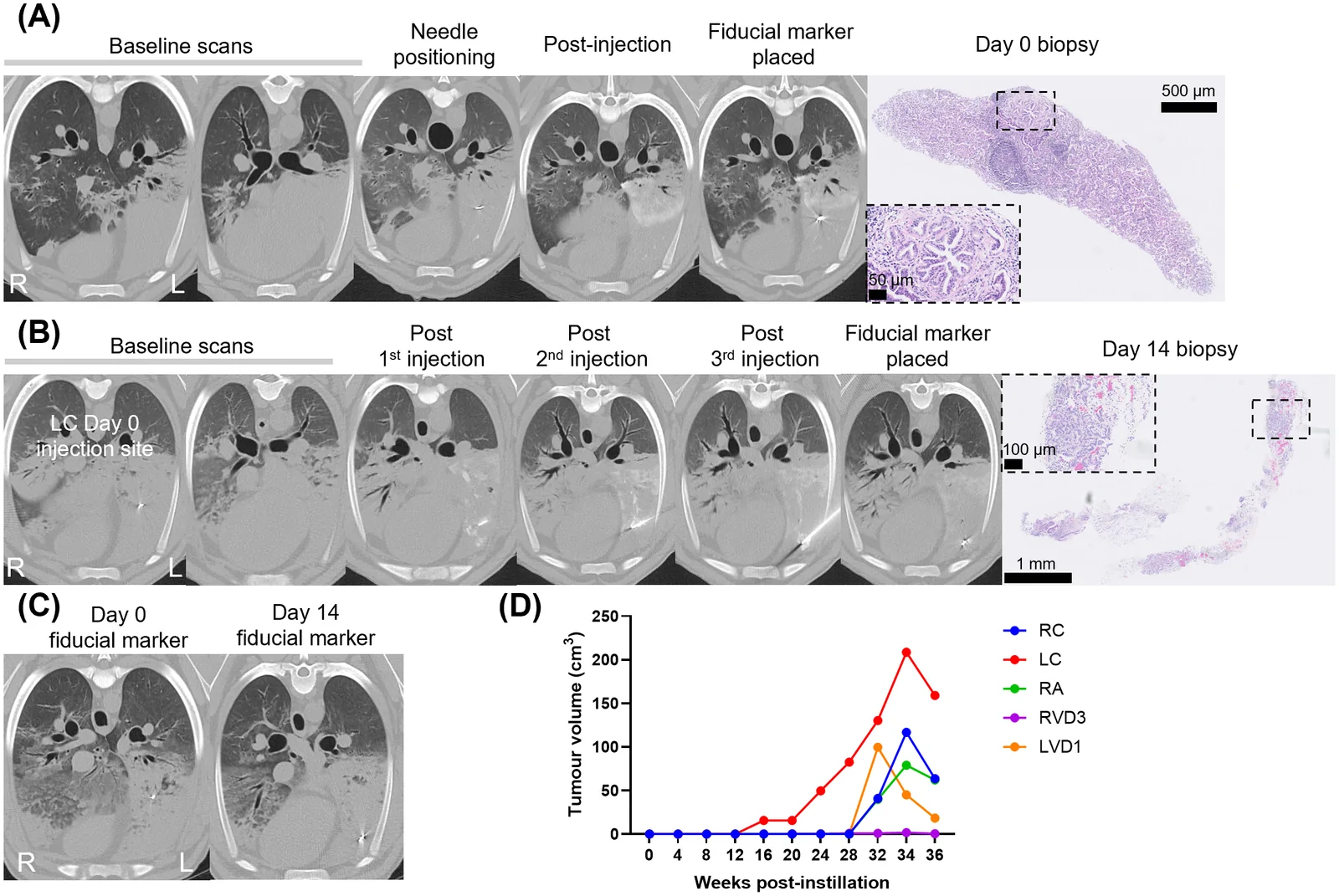
Intratumoural cisplatin injection experiments in a recovery sheep model. **(A)** Day 0 left cardiac (LC) injection; transthoracic intratumoural single bolus cisplatin injection, with fiducial marker placement post-injection in the LC lung tumour. Cisplatin/contrast mix can be seen in both LC and left ventral diaphragmatic 1 tumours post-injection. Representative H&E-stained biopsy obtained immediately prior to intratumoural cisplatin administration, demonstrating a well-differentiated epithelial neoplasm with lepidic growth patterns. L, left; R, right. **(B)** Day 14 LC injection; 3 transthoracic intratumoural cisplatin injections in different regions of the LC tumour, with fiducial marker placement post-injection. Cisplatin/contrast mix can be seen distributed throughout the LC tumour after the injections. Representative H&E-stained biopsy obtained immediately prior to day 14 intratumoural cisplatin administration, demonstrating neoplastic epithelial cells with associated stromal tissue and multifocal haemorrhage. L, left; R, right. **(C)** Day 28 CT scans of cisplatin-injected tumours taken on the day of euthanasia. Reduction in tumour volume after cisplatin treatment can be seen, in addition to the fiducial markers, which have remained in position. L, left; R, right. **(D)** Graph of tumour volumes up to the point of euthanasia. Day 0 cisplatin injection was performed at week 32; day 14 cisplatin injection was performed at week 34. RC, right cardiac; LC, left cardiac; RA, right apical; RVD3, right ventral diaphragmatic 3; LVD1, left ventral diaphragmatic 1.

3D tumour reconstructions were produced to assess tumour response to ITDD of cisplatin (**Figure 6D**). The LVD1 tumour, which received the majority of the day 0 cisplatin injection, exhibited a 55% decrease in volume 14 days post-injection. The volume of the LVD1 tumour at the point of euthanasia, 28 days after cisplatin injection, had decreased by 82%. The LC tumour also decreased in volume post-injection; by the day of euthanasia, the LC tumour had decreased in volume by 24%. The tumours present in the right cardiac, right apical and right ventral diaphragmatic 3 lobes had also reduced in volume by the end of the study.

Plasma was collected over the course of the 28-day experiment to track platinum levels in the blood post-intratumoural injection (**Figure 7A**). The day 0 LC tumour injection site had a quick release profile; platinum levels peaked immediately after injection with a value of 0.455 mg/L, decreasing to 0.122 mg/L 24 h post-injection. Platinum levels continued to reduce in the subsequent days, decreasing to their lowest level (0.036 mg/L) just prior to the day 14 cisplatin injection. On day 14, platinum levels in the plasma were analysed after the third fractionated injection into the LC tumour. The day 14 LC tumour injection sites exhibited a slower release profile; platinum levels peaked at 0.193 mg/L 5 min post-injection, decreasing to 0.091 mg/L 24 h post-injection. By the end of the study, platinum levels in the blood had not dropped to the levels seen prior to the cisplatin injections, and were recorded to be 0.045 mg/L.

**Figure 7:**
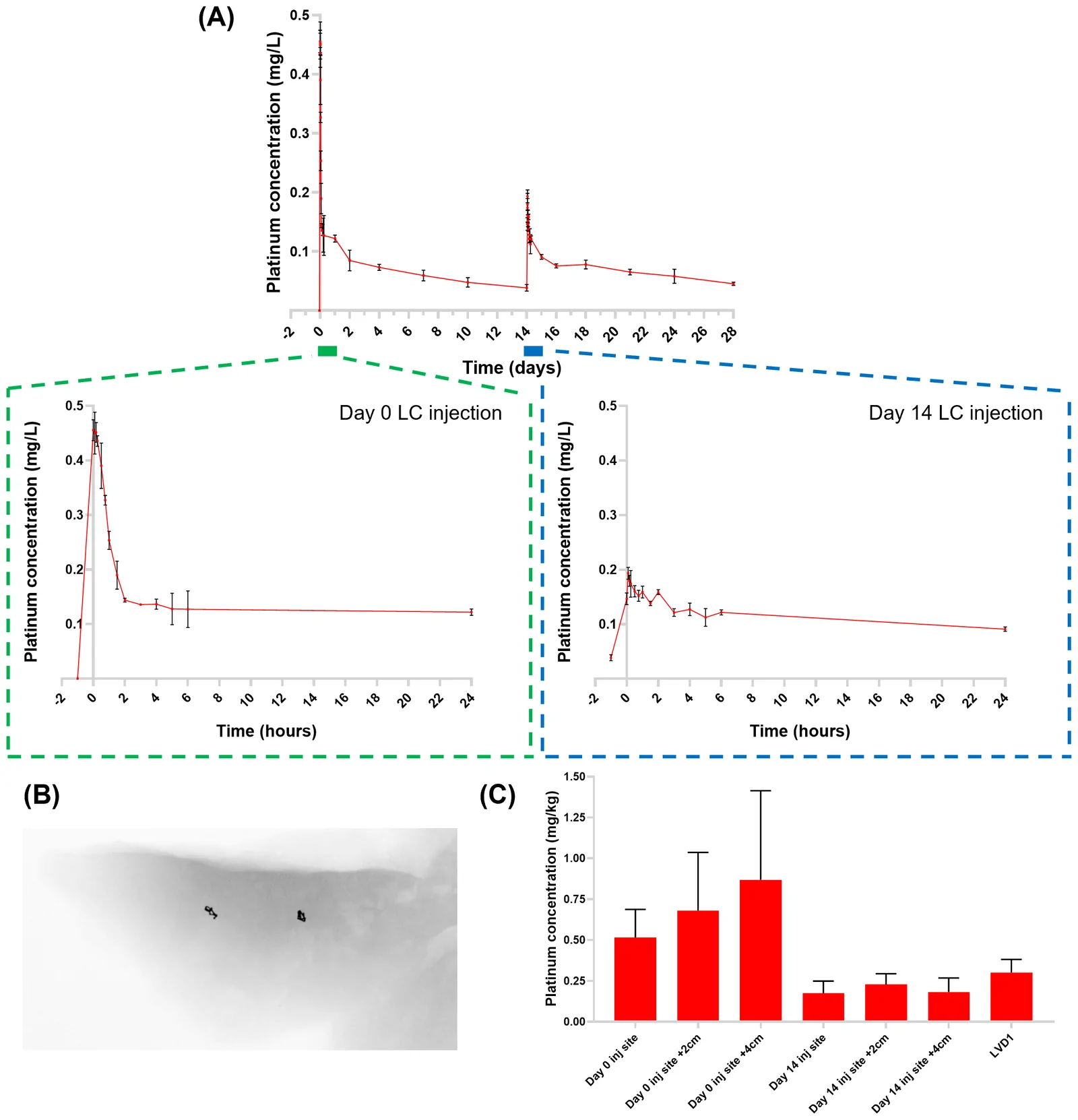
Plasma and tissue platinum levels post-cisplatin injection in a recovery sheep model. **(A)** Transthoracic injection of cisplatin was carried out in a recovery sheep protocol on 2 different days. The first injection (single bolus injection) was carried out on day 0 into the left cardiac tumour, with the second injection (3x fractions) performed 14 days later into the same tumour. Plasma platinum levels were analysed up to 28 days after the initial cisplatin injection (mean ± standard deviation, technical replicates = 2-6). LC, left cardiac. **(B)** Ex vivo fluoroscopic imaging of the left cardiac lung lobe. The platinum fiducial markers inserted on day 0 and day 14 can both be seen in the intact left cardiac lobe. **(C)** The levels of platinum in tissue acquired at post mortem was also analysed. Tissues analysed included the tumour injection sites at day 0 and day 14, tumour tissue at distances 2 cm and 4 cm away from these sites, and tumour tissue within left ventral diaphragmatic 1 (mean ± standard deviation, technical replicates = 3-4). LVD1, left ventral diaphragmatic 1; Inj, injection.

Following post mortem examination, tissues were taken to assess platinum concentrations at the tumour injection sites within the LC tumour, in tissues at 2 cm intervals from the injection sites, and in the LVD1 tumour. Fiducial markers were used to identify the cisplatin injection sites within the LC tumour (**Figure 7B**). The day 0 LC tumour tissues had the highest levels of platinum (**Figure 7C**).

### 3.5 Transthoracic injection targeting smaller tumour nodules

Previous experiments had tested methods for injecting cisplatin into large OPA tumours that had formed within the left and right cardiac lobes after catheter JSRV instillation. Here, we assessed the procedure for targeting smaller tumour nodules that arose in the diaphragmatic lobes after intra-parenchymal JSRV injection (**Figure 8**). The transthoracic injection of contrast agent was carried out successfully in a 22.88 cm^3^ tumour, with the contrast agent remaining within the confines of the tumour post-injection. Some hyperattenuation along the needle tract can be seen post-needle removal, likely representing minor intra-parenchymal haemorrhage.

**Figure 8:**
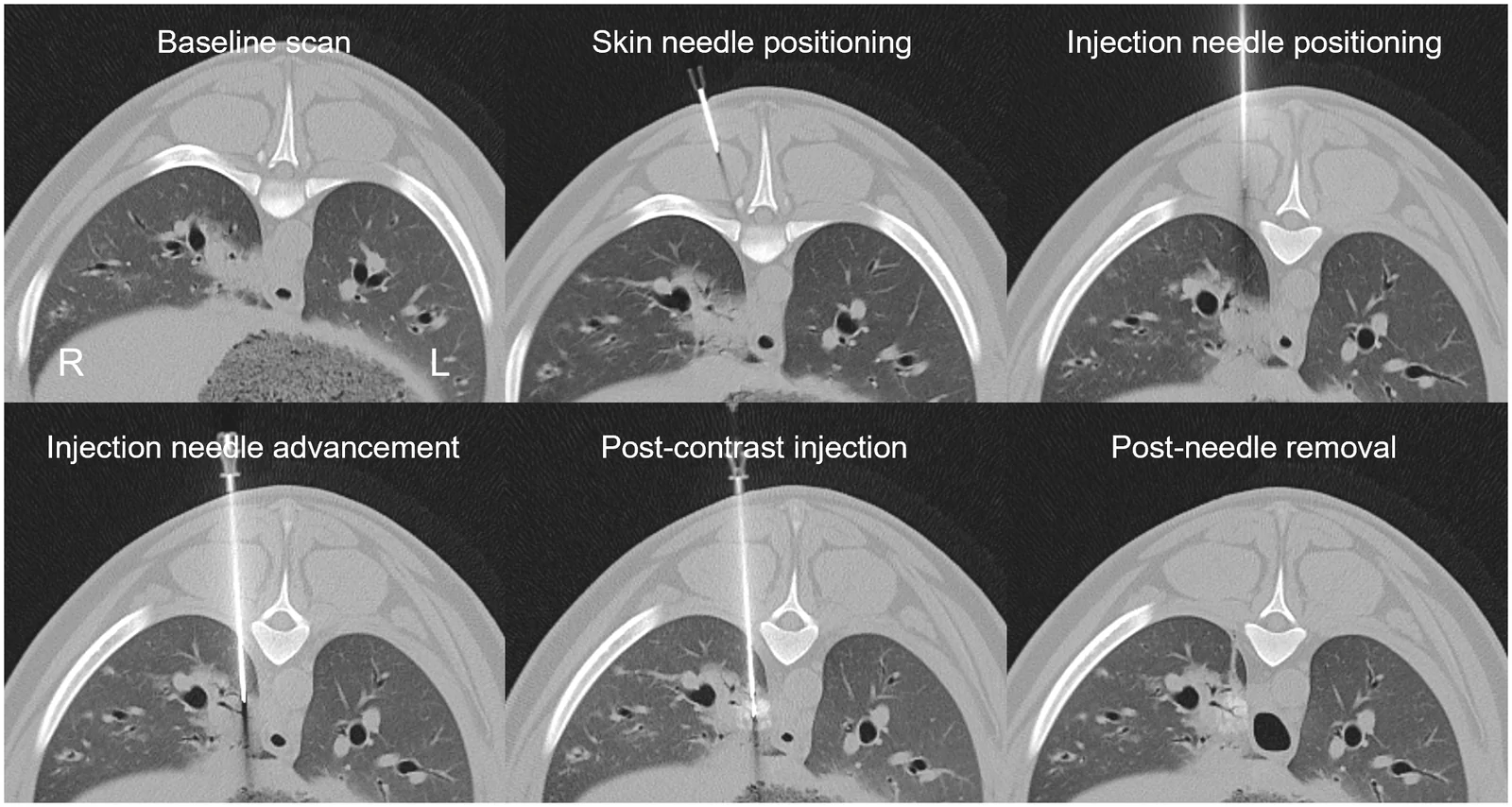
Contrast injection into small tumour nodules. Images represent axial CT scans taken from contrast injection into the right diaphragmatic tumour of sheep 85. Skin needle position was used to determine injection needle angulation. L, left; R, right.

## 4 Discussion

Previous large animal studies have explored locoregional cisplatin delivery in sheep; however, these investigations were performed in non-tumour-bearing animals and focused primarily on biomaterial-based drug delivery systems rather than tumour-directed pharmacology. In particular, cisplatin-loaded hyaluronate films have been evaluated in sheep as a post-surgical intrapleural therapy, demonstrating sustained local drug release and minimal systemic toxicity in the absence of malignant disease (23). While these studies provided important proof-of-concept for locoregional cisplatin delivery in a large animal setting, they did not address drug distribution, retention, or systemic exposure within lung tumours themselves.

In contrast to previous studies performed in healthy animals, the present work advances the field by delivering cisplatin directly into established lung tumours within our experimentally induced OPA model (68, 27). This comparative large animal platform enables evaluation of image-guided ITDD under physiologically relevant tumour-bearing conditions that cannot be replicated in healthy animals. Using this model, we developed both endobronchial and transthoracic ITDD techniques for the delivery of clinically relevant chemotherapeutic agents, approaches that have recently emerged as promising therapeutic strategies for NSCLC (8–11). We also established a fluoroscopic post-mortem protocol to identify the fiducial marker and injection site within excised OPA lesions, enabling accurate tissue sampling from the injection site and at defined distances from it for pharmacokinetic analysis. Furthermore, by evaluating both intravenous and intratumoural cisplatin administration, the model provides insight into systemic platinum exposure, tumour-associated platinum disposition, and the technical feasibility of image-guided therapeutic intervention, thereby extending previous large animal studies towards a more clinically relevant translational platform for intratumoural drug delivery.

Our results showed that both ITDD techniques could be conducted in our OPA model without evidence of major procedure-related complications, including haemorrhage, acute systemic adverse reactions or acute post-procedural deterioration. Post mortem identification of the fiducial marker and injection site was also shown to be successful in all cases. One sheep developed a moderate pneumothorax following needle removal during the transthoracic injection procedure. In humans, the leading cause of iatrogenic pneumothorax is transthoracic needle aspiration, usually carried out for the acquisition of biopsies (39). There are multiple technical factors that are associated with increased risk of transthoracic needle biopsy complications; these include the absence of preceding ipsilateral surgery procedures (40), a small oblique needle angle with the thoracic pleura (41), needle repositioning (42), the amount of normal aerated lung tissue crossed by the needle (43, 44), and the use of a trans-fissure approach (44). These factors were taken into account during the planning of transthoracic injections. The incidence of pneumothorax in human patients undergoing transthoracic procedures of lung lesions under CT guidance ranges from 8-64%, with an average of 20% (45). It has been reported that between 5-18% of biopsy procedures that result in pneumothorax require drainage (45). In our sheep, the pneumothorax was managed effectively during anaesthesia with intermittent thoracocentesis and did not affect the study outcomes.

Plasma and tissue platinum concentrations were assessed in sheep either after intravenous infusion of cisplatin (doses ranged from 118–124 mg) or following intratumoural cisplatin administration (doses ranged from 2 – 26.5 mg); the doses used were comparable to those described in previous reports in humans (10, 46–50). A standard clinical cisplatin formulation was used without modification for intravenous and intratumoural administration. Consequently, the observed distribution profiles reflect the behaviour of a formulation already used in clinical practice, rather than a specialised experimental preparation.

Intravenous administration led to similar plasma platinum profiles in each of the sheep; a rapid increase in plasma platinum levels was observed during the infusion period, peaking immediately at the end of the injection, followed by a rapid decline to achieve a relatively stable plateau by approximately 180 min post-infusion. In contrast, the tumours targeted in the non-recovery ITDD experiments exhibited varying cisplatin release profiles, with plasma platinum peaking at 5 min, 30 min and 2 h post-injection. Sheep 59 (transbronchial injection) had the quickest release profile, with high tissue platinum levels only seen at the injection site; these results suggest cisplatin may have entered the systemic circulation at the injection site, rather than being distributed throughout the tumour. As these results show, intratumoural therapy presents distinct pharmacokinetic challenges, as distribution and absorption can be more variable compared to systemic administration. Factors influencing drug distribution within a tumour and release into the systemic circulation are thought to include tumour volume, interstitial pressure, and the tumour vasculature itself (51); these features may explain the varying plasma platinum profiles seen in the ITDD sheep. Indeed, previous studies have shown that the retention of cisplatin within a tumour during ITDD can vary between advanced tumours in NSCLC patients (50).

Intravenous administration of approximately 120 mg cisplatin resulted in platinum concentrations of around 1–1.5 mg/kg in normal lung and tracheobronchial lymph node tissue. Following intratumoural administration, platinum was consistently detected within the targeted tumours despite the substantially lower cisplatin doses administered (2–26.5 mg). Platinum concentrations were highest at the injection site, with markedly lower concentrations detected in adjacent normal lung and regional lymph nodes, demonstrating preferential localisation of platinum to the targeted lesion. Although tumour platinum concentrations varied between animals, likely reflecting differences in tumour size, vascularity and intratumoural drug distribution, peak plasma platinum concentrations following ITDD remained approximately 100-fold lower than after intravenous administration; similar differences have been observed in human patients treated using these different methods (8). Although these findings suggest substantially lower systemic exposure following ITDD, this study was not designed to directly assess treatment-related toxicity. No clinical pathology, renal function, or histopathological toxicity assessments were performed to compare intravenous and intratumoural cisplatin administration. The tissue platinum measurements presented here were intended to characterise platinum distribution following each delivery strategy rather than provide a definitive quantitative comparison between intravenous and intratumoural administration. Given the proof-of-concept nature of the study, the small number of animals, and the differences in cisplatin dose and delivery route between treatment groups, larger studies will be required to undertake robust quantitative comparisons of tissue platinum distribution and treatment-associated toxicity.

Previous computer modelling studies have suggested that, rather than giving a single bolus of cisplatin to the centre of a lung tumour, it is instead more effective to distribute a cisplatin dose between optimally spaced locations throughout the tumour (52, 53). Through using multiple injection sites, it is thought that the total dose of cisplatin required could be reduced by 3–5 orders of magnitude, depending on tumour size (52, 54). To exhibit how our model could be used to assess these different intratumoural injection strategies, we conducted intratumoural cisplatin injection experiments using both single site cisplatin injection and fractionated protocols, in a recovery sheep study. This experiment was also performed to determine if we could perform intratumoural injections, biopsies and recovery anaesthesia using our OPA model. The single site cisplatin injection was performed on day 0, with the fractionated injections carried out 14 days later; the sheep was euthanised 28 days after the initial injection.

In this recovery sheep experiment, the injection volume of cisplatin was 25% of the tumour volume, calculated using reconstructions made through the CT scan images taken on the day of the injection. Basing cisplatin injection volume on the % of tumour volume has recently been outlined in other studies (46, 53). The cisplatin dose of 26.5 mg used in the recovery sheep experiment was also within the range of that used in previous studies on human patients with NSCLC (50). Prior publications have emphasised that rigorous assessment of drug delivery techniques necessitates observation of the drug with standard imaging techniques after injection (51); in the recovery sheep experiment, cisplatin was mixed with iohexol contrast agent to ensure that the distribution of injectate within the tumour could be observed on the immediate post-injection CT scans. As in the previous experiments, fiducial markers were placed to help identify the injection area at post mortem. Post-injection CT scans showed that while some of the cisplatin/contrast agent was present at the site of the first injection in the LC tumour, the majority of the injectate deposited in the tumour present in the LVD1 lobe. We believe that the tumour formed in the LVD1 lobe as a result of viral spread from the initial inoculation sites, as has occurred in our previous research on the model (68, 27). Studies have shown that tumour microenvironmental factors such as interstitial pressure and drug entry into blood vessels can lead to dramatic alterations in the intratumoural distribution of drugs post-injection (55); it is possible that one of these factors led to the majority of the injected cisplatin localising to the LVD1 tumour. As previously mentioned, prior studies have shown clear variation in cisplatin distribution post-ITDD in NSCLC tumours between patients (50), and even after proper needle placement, off-target drug distribution has occurred after intratumoural injection (56). Importantly, in our study, correct needle placement within the LC tumour was verified by fiducial marker localisation on post mortem fluoroscopic imaging, indicating that the observed drug redistribution was not a result of needle misplacement.

In contrast to the single bolus injection on day 0, the fractionated injections carried out on day 14 within the LC tumour led to drug disposition within the targeted tumour. Platinum levels were found to be highest within the LC tumour tissues around the day 0 injection site; it is possible that these tissues may have been exposed to cisplatin during both the day 0 and day 14 injections. As with the ITDD experiments performed with sheep 67 (transthoracic injection), 59 and 72 (both transbronchial injection), the injection sites targeted in sheep 94 exhibited varying cisplatin release profiles. The day 0 LC tumour injection site had a quick release profile, with platinum levels peaking after injection had finished. The day 14 LC tumour injection sites exhibited a slower release profile, with platinum levels peaking 5 min post-injection. Again, variations in interstitial pressure and the tumour vasculature between the injection sites may explain the different release profiles (51). Total plasma platinum concentrations had not returned to pre-treatment levels by the time of euthanasia. Because platinum concentrations were measured using ICP-MS, these data reflect total platinum rather than intact cisplatin specifically. Although only two intratumoural injections were administered during the recovery experiment, prolonged persistence of platinum-containing species is biologically plausible. Cisplatin undergoes rapid aquation and extensive protein binding following administration, which can strongly influence distribution and pharmacokinetics (57, 58). Consistent with this, previous studies have reported significantly elevated blood platinum concentrations in human patients up to 20 years following completion of cisplatin-based chemotherapy regimens (59–62). In the present study, following ITDD, the persistence of plasma platinum may also reflect the retention of platinum-containing species within the tumour microenvironment and subsequent sustained systemic release.

CT imaging is commonly used to assess tumour response to treatment in oncology, focusing on changes in size to determine if a tumour is shrinking, growing, or stable. Initial work published in 1976 (63) eventually led to the Response Evaluation Criteria in Solid Tumors guidelines, which set specific thresholds for size changes that rely on the measurement of alterations in lesion diameter (64). Radiological measurement of tumour burden has advanced with imaging and computational technology. Volumetric assessment provides significant advantages over traditional linear measurements for evaluating response to treatment, as it can greatly reduce measurement variance, enhance statistical power per subject, and has greater power to accurately detect changes in tumour size (65, 66). Here, 3D tumour reconstructions were produced to assess how the tumours responded to cisplatin injection. The LVD1 tumour which, as observed through the CT scans was the tumour in which the majority of the day 0 cisplatin deposited, exhibited the largest decrease in size, reducing in volume by 82% by the end of the study. The LC tumour, in which the day 14 cisplatin injection deposited, also exhibited a 24% decrease in volume by the end of the study. Tumours not targeted by our protocol also reduced in volume. In the non-recovery ITDD experiments (sheep 59, 67, and 72), platinum was detected in the tissue of non-targeted tumours. It is therefore possible that the volume reduction observed in the non-targeted tumours within the recovery sheep experiment may be due to cisplatin entering the systemic circulation, and depositing in these other tumours, after intratumoural injection.

The tumours targeted in the cisplatin ITDD experiments within this study ranged in volumes from 67.57 cm^3^ to 222.3 cm^3^. Patients eligible for ITDD can present with lesions of varying sizes; a recent study, assessing the effects of intratumoural cisplatin treatment for advanced NSCLC, included patients with tumours ranging in volume from 0.95 cm^3^ to 229.95 cm^3^, with an average of 14.1 cm^3^ (54). To take into account the smaller tumours that can be targeted with intratumoural injection, we also performed experiments showing the capability of our model to test ITDD techniques targeting smaller tumour nodules, performing the transthoracic injection of contrast agent into a tumour 22.88 cm^3^ in volume. This technique was carried out successfully, with the contrast agent seen to remain within the confines of the tumour post-injection. This lays the technical groundwork to expand our current model to include a wider range of clinically relevant cases.

Several limitations of this study should be acknowledged. The recovery component was performed in a single animal and therefore should be regarded as a proof-of-concept assessment of repeated intratumoural drug delivery, tumour biopsy acquisition, CT monitoring, and recovery anaesthesia within the OPA model. Consequently, inter-animal variability in platinum disposition and tumour response could not be assessed, and conclusions regarding the biological effects of intratumoural cisplatin administration should be interpreted cautiously pending confirmation in larger studies. More broadly, the relatively small number of animals and procedures performed in the non-recovery protocols means that the present study demonstrates procedural feasibility rather than establishing procedural safety. Although both transthoracic and transbronchial ITDD approaches were successfully completed in all animals, one sheep developed a moderate pneumothorax following transthoracic needle withdrawal that required intraoperative thoracocentesis. Further studies involving larger numbers of animals will be required to more comprehensively evaluate variability in treatment response, define complication rates, and characterise the procedural risks associated with these techniques.

Although the present work utilised our experimentally induced tumour model to provide controlled tumour location, timing of tumour development, and standardised assessment of therapeutic interventions, naturally occurring OPA cases also represent an important comparative model with future translational potential (21, 67). However, the use of spontaneous cases presents several practical and scientific challenges. Early disease is difficult to identify because there is currently no sensitive preclinical diagnostic test, with transthoracic ultrasonography typically detecting only more advanced lesions. Consequently, naturally occurring cases are poorly suited to longitudinal therapeutic studies investigating early tumour development, which is often the stage of greatest relevance for therapeutic intervention in human lung cancer. Furthermore, transport of affected sheep from their farm of origin to a research facility can precipitate clinical deterioration, potentially necessitating euthanasia before therapeutic studies can be undertaken. We therefore view experimentally induced and naturally occurring OPA as complementary rather than competing models. The experimentally induced model addresses questions that cannot readily be investigated in spontaneous disease, including longitudinal assessment of tumour development, optimisation of image-guided therapeutic interventions, and controlled evaluation of treatment response from earlier stages of disease. Conversely, naturally occurring cases, where suitable animals can be identified, may provide an opportunity to evaluate established therapeutic approaches within a spontaneous disease setting and enhance their clinical relevance. Future studies integrating experimentally induced OPA, naturally occurring OPA and human clinical datasets may further strengthen the translational value of this comparative oncology platform and accelerate the development of lung cancer therapies.

## 5 Conclusion

Intratumoural drug delivery is a promising strategy to enhance therapeutic efficacy while minimising systemic toxicity; however, further preclinical and clinical studies are required before these approaches can be more widely adopted in the treatment of lung cancer. There is a growing consensus that translational research using comparative large animal models, which closely mimic human anatomy, tumour biology and treatment response, are crucial for advancing therapies toward clinical use. The results presented here establish the experimentally induced ovine lung cancer model as a clinically relevant large animal platform for the development and evaluation of intratumoural therapeutic delivery strategies. By enabling longitudinal tumour monitoring, image-guided intervention, repeat biopsy acquisition, and assessment of systemic and tissue platinum exposure, the model provides a unique opportunity to bridge preclinical therapeutic development and human lung cancer treatment.

## Data Availability

The original contributions presented in the study are included in the article/**Supplementary Material**. Further inquiries can be directed to the corresponding author.

## Supplementary material

Supplementary Figure 1 Transbronchial intralesional injection of cisplatin in sheep 59. Axial CT images showing bronchoscope and needle placement within the left cardiac lung lobe lesion, followed by fiducial marker placement.
