# Supplementary material for: A large animal lung cancer model for the evaluation of intratumoural cisplatin delivery strategies
Source: Front Oncol. 2026 Oct 9;16:1906629. doi: 10.3389/fonc.2026.1906629 (PMC13691547; doi:10.3389/fonc.2026.1906629)
Supplement: Supplementary file 1 [file Table1.docx]

**Supplementary table 1.** Techniques used to provide ovine anaesthesia and analgesia for short-term (6-7 h), non-recovery, intravenous cisplatin studies. * Optional drugs provided to 1 sheep only. IV, intravenous.

| **Phase** | **Drug** | **Manufacturer** | | **Dose (mg/kg)** | **Route** |
| --- | --- | --- | --- | --- | --- |
| **Pre-anaesthetic medication** | Medetomidine | ‘Medetor’; 1 mg/ml, Virbac Ltd | | 0.005 | IV |
|  | Ketamine | ‘Ketamidor’; 100 mg/ml, Chanelle Vet Ltd | | 1 | IV |
|  | Midazolam* | ‘Hypnovel’; 10 mg/2 ml, Roche | | 0.25 | IV |
|  | Acepromazine* | ‘Acesedate’; 2 mg/ml, Zoetis | | 0.05 | IV |
|  | | |  | | |
| **Induction** | Propofol | ‘Propofol’; 1% w/v, Fresenius Kabi | | To effect (e.g., 2-5) | IV |
|  | | |  | | |
| **Maintenance** | Isoflurane | ‘Isofane’; Abbot Animal Health | | 1.1-1.7% | inhaled |
|  | Ketamine | ‘Ketamidor’; 100 mg/ml, Chanelle Vet Ltd | | 0.01 mg/kg/min | IV |
|  | Midazolam | ‘Hypnovel’; 10 mg/2 ml, Roche | | 0.2 mg/kg/h | IV |
|  | | |  | | |
| **Fluid therapy** | 0.9% sodium chloride | ‘Aqupharm No 1’; Animalcare | | 5 ml/kg/h | IV |
