# Supplementary material for: A large animal lung cancer model for the evaluation of intratumoural cisplatin delivery strategies
Source: Front Oncol. 2026 Oct 9;16:1906629. doi: 10.3389/fonc.2026.1906629 (PMC13691547; doi:10.3389/fonc.2026.1906629)
Supplement: Supplementary file 5 [file Table5.docx]

| **Sheep** | **Sex** | **Age (months)** | | **Weight (kg)** |
| --- | --- | --- | --- | --- |
| 35 | Female | 2 | 27 | |
| 39 | Female | 2 | 30 | |
| 41 | Female | 2 | 25 | |
| 67 | Male | 2 | 21 | |
| 59 | Female | 2 | 21 | |
| 72 | Female | 2 | 21 | |
| 94 | Female | 2 | 34 | |
| 85 | Female | 2 | 27 | |

**Supplementary table 5.** Sex, age, and weight of sheep at the time of JSRV administration.
