# Supplementary material for: A large animal lung cancer model for the evaluation of intratumoural cisplatin delivery strategies
Source: Front Oncol. 2026 Oct 9;16:1906629. doi: 10.3389/fonc.2026.1906629 (PMC13691547; doi:10.3389/fonc.2026.1906629)
Supplement: Supplementary file 6 [file Table6.docx]

**Supplementary table 6.** Blood collection time points relative to terminal intravenous cisplatin administration in sheep 35, 39 and 41.

| **Blood sample acquisition intervals** |  | 15 minutes | | 5 minutes | | | 15 minutes | | 30 minutes | | | 1 hour | | | | 30 minutes |
| --- | --- | --- | --- | --- | --- | --- | --- | --- | --- | --- | --- | --- | --- | --- | --- | --- |
| **Minutes** | Baseline | -15 | 0 | 5 | 10 | 15 | 30 | 45 | 60 | 90 | 120 | 180 | 240 | 300 | 360 | 390 |
