# Supplementary material for: A large animal lung cancer model for the evaluation of intratumoural cisplatin delivery strategies
Source: Front Oncol. 2026 Oct 9;16:1906629. doi: 10.3389/fonc.2026.1906629 (PMC13691547; doi:10.3389/fonc.2026.1906629)
Supplement: Supplementary file 7 [file Table7.docx]

**Supplementary table 7.** Blood collection time points relative to transthoracic cisplatin administration in sheep 67.

| **Blood sample acquisition intervals** |  | 5 minutes | | | | 15 minutes | | | 30 minutes | | 1 hour | | | | | 30 minutes |  |  |  |  |  |  |  |  |  |  |  |  |  |  |  |  |
| --- | --- | --- | --- | --- | --- | --- | --- | --- | --- | --- | --- | --- | --- | --- | --- | --- | --- | --- | --- | --- | --- | --- | --- | --- | --- | --- | --- | --- | --- | --- | --- | --- |
| **Minutes** | Baseline | 0 | 5 | 10 | 15 | 30 | 45 | 60 | 90 | 120 | | 180 | 240 | 300 | 360 | 390 |  |  |  |  |  |  |  |  |  |  |  |  |  |  |  |  |
|  |  |  |  |  |  |  |  |  |  |  | |  |  |  |  |  |  |  |  |  |  |  |  |  |  |  |  |  |  |  |  |  |
