# Supplementary material for: A large animal lung cancer model for the evaluation of intratumoural cisplatin delivery strategies
Source: Front Oncol. 2026 Oct 9;16:1906629. doi: 10.3389/fonc.2026.1906629 (PMC13691547; doi:10.3389/fonc.2026.1906629)
Supplement: Supplementary file 8 [file Table8.docx]

**Supplementary table 8.** Blood collection schedule relative to transthoracic cisplatin administration on days 0 and 14 in the recovery experiment (sheep 94).

| **Day 0** |  |  | | | |  | | |  | |  | | | |
| --- | --- | --- | --- | --- | --- | --- | --- | --- | --- | --- | --- | --- | --- | --- |
| **Blood sample acquisition intervals** |  | 5 minutes | | | | 15 minutes | | | 30 minutes | | 60 minutes | | | |
| **Minutes** | Baseline | 0 (after injection) | 5 | 10 | 15 | 30 | 45 | 60 | 90 | 120 | 180 | 240 | 300 | 360 |
|  |  |  |  |  |  |  |  |  |  |  |  |  |  |  |
| **Days** | 1 | 2 | 4 | 7 | 10 |  |  |  |  |  |  |  |  |  |
|  |  |  |  |  |  |  |  |  |  |  |  |  |  |  |
| **Day 14** |  |  |  |  |  |  |  |  |  |  |  |  |  |  |
| **Blood sample acquisition intervals** |  | 5 minutes | | | | 15 minutes | | | 30 minutes | | 60 minutes | | | |
| **Minutes** | Baseline | 0 (after injection) | 5 | 10 | 15 | 30 | 45 | 60 | 90 | 120 | 180 | 240 | 300 | 360 |
|  |  |  |  |  |  |  |  |  |  |  |  |  |  |  |
| **Days** | 15 | 16 | 18 | 21 | 24 | 28 |  |  |  |  |  |  |  |  |
